# Cancer-associated fibroblast-derived gene signature discriminates distinct prognoses by integrated single-cell and bulk RNA-seq analyses in breast cancer

**DOI:** 10.18632/aging.205817

**Published:** 2024-05-09

**Authors:** Zhou Fang, Yi-Ling Han, Zhi-Jie Gao, Feng Yao

**Affiliations:** 1 Department of Breast and Thyroid Surgery, Renmin Hospital of Wuhan University, Wuhan, Hubei, P. R. China; 2 Center for Reproductive Medicine, Renmin Hospital of Wuhan University, Wuhan, Hubei, P. R. China

**Keywords:** breast cancer, cancer-associated fibroblasts, single-cell RNA-seq analyses

## Abstract

Background: Cancer-associated fibroblasts (CAFs) are one of the most predominant cellular subpopulations in the tumor stroma and play an integral role in cancer occurrence and progression. However, the prognostic role of CAFs in breast cancer remains poorly understood.

Methods: We identified a number of CAF-related biomarkers in breast cancer by combining single-cell and bulk RNA-seq analyses. Based on univariate Cox regression as well as Least Absolute Shrinkage and Selection Operator (LASSO) regression analysis, a novel CAF-associated prognostic model was developed. Breast cancer patients were grouped according to the median risk score and further analyzed for outcome, clinical characteristic, pathway activity, genomic feature, immune landscape, and drug sensitivity.

Results: A total of 341 CAF-related biomarkers were identified from single-cell and bulk RNA-seq analyses. We eventually screened eight candidate prognostic genes, including *CERCAM*, *EMP1*, *SDC1*, *PRKG1*, *XG*, *TNN*, *WLS*, and *PDLIM4*, and constructed the novel CAF-related prognostic model. Grouped by the median risk score, high-risk patients showed a significantly worse prognosis and exhibited distinct pathway activities such as uncontrolled cell cycle progression, angiogenesis, and activation of glycolysis. In addition, the combined risk score and tumor mutation burden significantly improved the ability to predict patient prognosis. Importantly, patients in the high-risk group had a higher infiltration of M2 macrophages and a lower infiltration of CD8^+^ T cells and activated NK cells. Finally, we calculated the IC50 for a range of anticancer drugs and personalized the treatment regimen for each patient.

Conclusion: Integrating single-cell and bulk RNA-seq analyses, we identified a list of compositive CAF-associated biomarkers and developed a novel CAF-related prognostic model for breast cancer. This robust CAF-derived gene signature acts as an excellent predictor of patient outcomes and treatment responses in breast cancer.

## INTRODUCTION

Breast cancer stands as the most prevalent malignant tumor in women worldwide. According to the latest statistics, it is projected that in 2023 there will be 297,790 new cases accounting for 31% of female cancers and 43,170 deaths accounting for 15% of female deaths [1, 2]. All these data underscore an urgent need to establish new and effective strategies for the diagnosis and treatment of breast cancer. Due to the significant heterogeneity of breast cancer, there is a growing interest in the clinical realm towards personalized and precise treatment approaches [3, 4]. The pursuit of identifying novel prognostic biomarkers and promising therapeutic targets is recognized as a crucial avenue to address this imperative.

The tumor microenvironment (TME) is an intricate ecosystem composed of malignant cells, a variety of infiltrating immune cells (lymphocytes and myeloid cells), and stromal cells intertwined with noncellular components [5]. Cancer-associated fibroblasts (CAFs) play a pivotal role in the progression of tumors [6]. As a major component of the stroma, CAFs enhance cancer cell proliferation, immune rejection, and treatment resistance by secreting growth factors and inflammatory ligands [7]. For example, CAFs could secrete vascular endothelial growth factor (VEGF) to regulate the tumor vascular network [8], and interleukin 6 (IL-6) to foster the differentiation of myeloid-derived suppressor cells (MDSCs) and suppress cytotoxic T cells [9]. Furthermore, the secretion of *CXCL12* by CAFs upregulates the anti-apoptotic proteins *Bcl-2* and *Survivin*, potentially contributing to therapy resistance in breast cancer [10]. CAFs can also shape the extra cellular matrix by secreting collagen, fibrinolytic proteins, hyaluronic acid, and laminin, forming a barrier to drugs or therapeutic immune cell penetration. This impediment prevents deep penetration of drugs and immune cells into tumor tissues, thereby diminishing the efficacy of tumor therapy. The modulation of CAFs or overcoming their barrier effect represents a novel approach in tumor therapy [11, 12]. Nevertheless, there is still a deficiency of breast cancer prognostic models based on CAF-specific gene markers to date.

Phenomenal advances in single-cell RNA sequencing (scRNA-seq) technologies have allowed us to deeply analyze the complex cellular composition inside tumors. Here, we conducted a reanalysis a publicly available breast cancer single-cell RNA-seq cohort containing 26 samples [13]. The specific gene markers of CAFs in breast cancer were interrogated by Wilcoxon rank-sum test algorithm at single-cell resolution. Additionally, combining multiple deconvolution algorithms and Weighted Gene Co-expression Network Analysis (WGCNA), we also identified a group of CAFs marker genes. The overlapped compartment of above markers was selected as the final CAF-associated marker genes. Next, we performed univariate Cox regression and least absolute shrinkage and selection operator (LASSO) regression analyses in The Cancer Genome Atlas (TCGA) breast cancer cohort to build a CAF-related prognostic model. Based on the median risk score, the breast cancer samples were classified into high and low-risk groups. Reasonably, patients in the high-risk group showed markedly inferior outcomes. In addition, the potential predictive power of the prognostic model was validated in several separate validation sets. More importantly, pathway activity, mutational profile, and immune status exhibited dramatic differences between high- and low-risk groups. High-risk patients have disturbed cell cycle progression and demonstrate epithelial mesenchymal transition. In addition, high-risk patients were characterized by more M2 macrophage infiltration and less CD8^+^ T cell. Finally, we focused on the response to chemotherapeutic agents of patients in different risk groups and interrogated the sensitivities of several anti-cancer drugs among diverse risk group patients. To summarize, we combined scRNA-seq and bulk RNA-seq to construct a robust CAF-related prognostic model, which can be used to guide the prognosis and treatment of breast cancer patients.

## MATERIALS AND METHODS

### Single-cell transcriptome analysis

In our study, we initially obtained scRNA-seq data from 26 breast cancer samples from the GEO database (GSE176078) [13]. We performed unsupervised clustering of the individual cells using the read count matrix as input, employing the Seurat package (v4.1.1) in R (v4.1.3) [14]. Stringent quality control criteria were implemented, primarily focusing on the number of detected genes and the proportion of mitochondrial gene counts per cell.

Initially, we filtered out cells with fewer than 200 detected genes and cells with more than 15% mitochondrial gene counts. We used the Harmony algorithm to integrate the multi-sample data and correct batch effects [15]. Subsequently, dimension reduction clustering and differential expression analysis were performed following the Seurat-guided tutorial. Principal component analysis (PCA) and uniform manifold approximation and projection (UMAP) dimension reduction were carried out using the top 20 principal components. Cell cluster annotations were based on canonical gene markers.

### Weighted gene co-expression network analysis

We used the WGCNA package to obtain genes most related to CAFs content [16]. Samples were clustered to ascertain the overall relevance of all samples in the dataset, and outliers were excluded. The soft thresholding power β was chosen based on the lowest power for which the scale-free topology fit index reached a high value. The minimum gene number/module was set to 50 and, finally, 11 modules were generated. Next, we undertook correlation analyses between modules and traits to find the most relevant modules for CAFs content.

### Collection of public datasets

RNA-sequencing expression matrix and clinical information of breast cancer samples and para cancerous tissues from The Cancer Genome Atlas (TCGA) database were downloaded from UCSC Xena (https://xena.ucsc.edu/). Three additional independent datasets (GSE20685, GSE37751 and GSE58812) were obtained from the GEO database (https://www.ncbi.nlm.nih.gov/geo/) [17–19]. We downloaded somatic mutation data from Genomic Data Commons (GDC, https://portal.gdc.cancer.gov/). Somatic mutation data sorted in the form of Mutation Annotation Format (MAF) were analyzed and then used to calculate Tumor mutation burden (TMB) using the R package maftools [20].

### Construction and validation of a CAF-related prognostic signature

First, 1205 genes related to CAFs were collected from single-cell transcriptome analysis and 487 yellow module genes from WGCNA. Taking the intersection of the two yielded 341 candidate CAF-related genes for breast cancer. To obtain CAF-related genes that could construct a prognostic signature, univariate Cox regression and least absolute shrinkage and selection operator (LASSO) regression analyses were carried out. We eventually obtained 8 genes, including *CERCAM*, *EMP1*, *SDC1*, *PRKG1*, *XG*, *TNN*, *WLS*, and *PDLIM4*, and constructed a CAF-related prognostic model based on these genes. To group the breast cancer patients, the risk score of each breast cancer patient in the training set was calculated according to the following formula:


Risk score=∑ni=∑(Coefi×xi)

The breast cancer patients were then categorized into the high-risk and low-risk groups according to the median of risk score. The predictive sensitivity of the risk score was painted via the R package survival ROC for estimation [21]. The model effectiveness was evaluated in the validation set using the same coefficient and cutoff values that were used in the training set.

### Biological functional analysis between high/low-risk group patients

The DESeq2 R package was used to perform differentially expressed genes (DEGs) analysis. DEGs were determined with a cutoff of an adjust *p*-value of less than 0.05 and |Log2 fold change| greater than 1 [22]. The clusterProfiler R package was used to perform gene set enrichment analysis (GSEA) [23]. With the use of Fisher’s exact test, those with false discovery rate FDR-corrected *p*-values of less than 0.05 were regarded as marked indicators. Single sample gene set enrichment analysis was performed via the R package GSVA [24]. Gene signatures of recurrent cancer cell states were collected from the previous study. The ITH score was calculated using the DEPTH R package [25].

### Tumor immune microenvironment in breast cancer patients

To study the infiltration of immune cells, we used TIMER2.0, an efficient algorithm for predicting immune cell infiltration of bulk tumor gene expression data (http://timer.cistrome.org/). In addition, we collected series of tumor immunomodulators from the literatures and calculated the correlation of risk score with them.

### Predicting drug responses and immunotherapy sensitivity

We used the R package oncoPredict to assess the predictive ability of risk score chemotherapeutic agents by calculating patients IC50 for various common chemotherapeutic agents. The Wilcoxon rank test was then used to compare the difference in IC50 between the high/low-risk groups.

### Univariate and multivariable Cox regression

We performed univariate Cox regression on breast cancer patients with gene expression and overall survival. Multivariate Cox regression was used to evaluate independent risk factors in the same cohort. Genes and factors with a false discovery rate (FDR) <0.05 were considered statistically associated with patient survival. The results of univariate and multivariate Cox regression were acquired and visualized by using the R package forestplot.

### Establishment of the nomogram

This study used the Cox regression model along with the R package rms to build an OS prediction nomogram that set 1-, 2-, 3-, and 5-year OS as the endpoints.

### Statistical analysis

All statistical analyses were performed using R version 4.1.3 (https://www.r-project.org/) and its adequate packages. Statistical significance was set at *p* ≤ 0.05.

### Availability of data and materials

The datasets used and/or analyzed during the current study are available from the corresponding author on reasonable request.

## RESULTS

### Interrogating the cellular constitution of breast cancer at single-cell resolution

**Figure 1:**
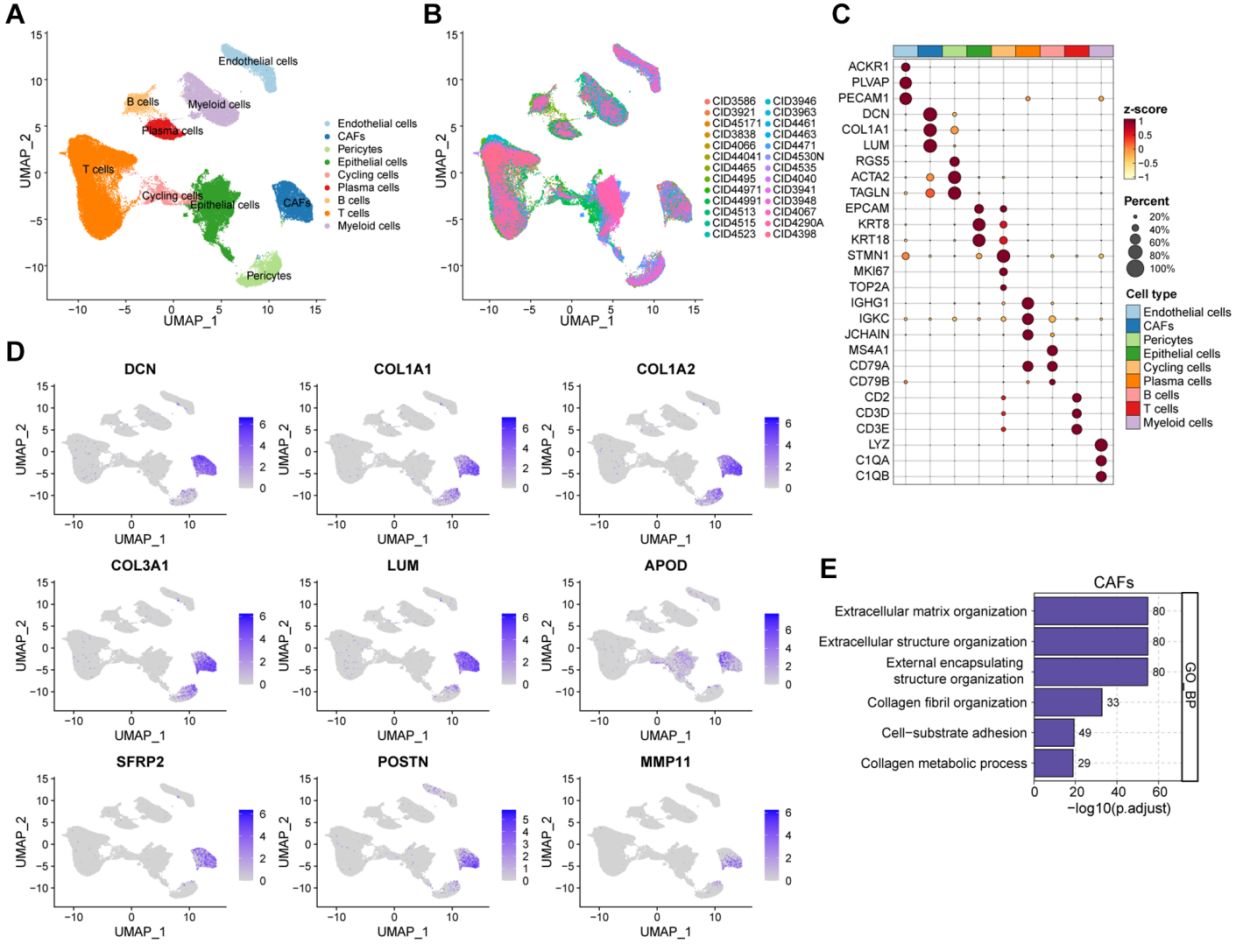
**Interrogating the cellular constitution of breast cancer at single-cell resolution.** (**A**, **B**) UMAP plot showing the major cell subpopulations in breast cancer. (**C**) Bubble heatmap showing expression levels of selected signature genes in breast cancer. Dot size indicates fraction of expressing cells, colored based on normalized expression levels. (**D**) Feature plots to further identify various CAFs, based on the expression levels of marker genes. (**E**) GO enrichment of CAFs signature genes.

In order to meticulously investigate the cellular constitution of breast cancer at single-cell resolution and identify cell markers of CAFs, we re-analyzed the scRNA-seq data of tumors from 26 breast cancer samples. Firstly, we integrated these data and corrected the potential batch effects through Harmony algorithm. Following rigorous quality control and data filtering, data of 29733 genes within 85408 cells was obtained. Three major compartments in the TME of breast cancer, including the immune subset, the stromal subset, and the epithelial subset were identified (Figure 1A). UMAP visualization showed that scRNA-seq data from different samples were integrated and mixed uniformly (Figure 1B). Using canonical lineage markers, we annotated each cell subpopulation among three main cellular subsets as epithelial cells, T cells, B cells, plasma cells, myeloid cells, endothelial cells, pericytes, cycling cells, and CAFs (Figure 1C, Supplementary Table 1). For instance, the immune subset consisted of T cells which were identified by expressions of *CD2*, *CD3D* and *CD3E*, B cells with high *MS4A1*, *CD79A*, and *CD79B* expressions, plasma cells with high *IGHG1*, *IGKC*, and *JCHAIN* expressions, and myeloid cells which were identified by significant expressions of *LYZ*, *C1QA* and *C1QB* (Figure 1C). Additionally, we found several stromal subpopulations including endothelial cells, pericytes, and CAFs. We annotated the endothelial cells due to the unique expression of *ACKR1*, *PLVAP* and *PECAM1*, as well as pericytes with expressions of *RGS5*, *ACTA2*, *TAGLN* (Figure 1C, 1D). Moreover, with a special focus on the CAFs, we observed CAFs showed significant expressions of *DCN*, *COL1A1* and *LUM*, which were believed to be uniquely expressed proteins on the surface of CAFs and played a role in the development and function of CAFs. As expected, the Gene Ontology (GO) enrichment analysis exhibited that CAFs gene markers were enriched in pathways including extracellular matrix organization, extracellular structure organization and collagen fibril organization (Figure 1E). Collectively, we interrogated the cellular constitution of breast cancer in detail at single-cell resolution and identified cellular marker genes of each subpopulation, especially for CAFs.

### Screening for CAF-related genes by WGCNA in breast cancer

**Figure 2:**
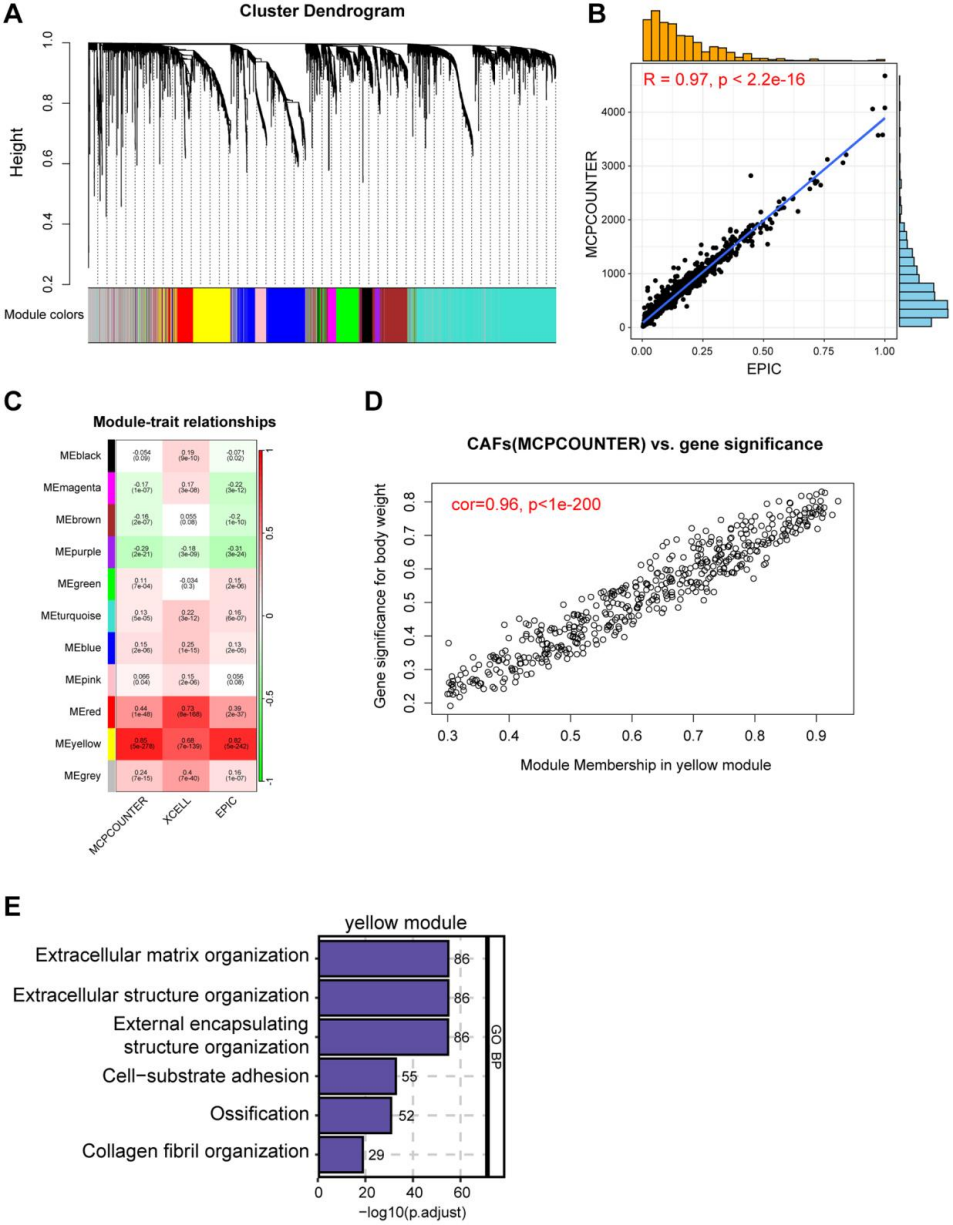
**Screening for CAF-related genes by WGCNA in breast cancer.** (**A**) The cluster dendrogram constructing the gene modules and module merging. (**B**) Correlation plot of infiltration of CAFs by EPIC and MCPCOUNTER. (**C**) Correlation analysis of modules with traits yielded 11 modules, with the yellow module considered to be the most relevant module for CAFs. (**D**) Scatter plot between the yellow module and MCPCOUNTER. (**E**) GO enrichment of yellow module genes.

To comprehensively anatomize CAF-related biomarkers, we implemented WGCNA in the bulk RNA-seq dataset of breast cancer. Initially, following the removal of outliers from the TCGA samples, five were selected as the optimal soft-threshold power and 11 modules were identified by WGCNA algorithm (Figure 2A and Supplementary Figure 1A–1C). We compared data from three immune infiltration algorithms, including MCPCOUNTER, EPIC, and XCELL, as we expected, there was excellent congruity on the percentage of CAFs among these data (Figure 2B and Supplementary Figure 1D, 1E). In order to pinpoint the key modules of WGCNA associated with CAFs, we separately calculated the correlation of the three types of immune infiltration data with the modules. As shown in Figure 2C, the yellow module exhibited the highest correlation to the deconvolution result according to the MCPCOUNTER and EPIC, and equal importance in the XCELL result (Supplementary Table 2). Collectively, we designated the gene in the yellow module as a potential biomarker for CAFs in breast cancer (Figure 2D and Supplementary Figure 1F, 1G). In addition, astonishingly, the results of the GO enrichment analysis of the yellow module genes were highly consistent with the enrichment analysis of the CAFs marker genes identified by scRNA-seq data (Figure 2E). In conclusion, our analysis revealed a significant correlation between the yellow module and CAFs in breast cancer through the application of WGCNA.

### Construction of a CAF-related prognostic signature in breast cancer

**Figure 3:**
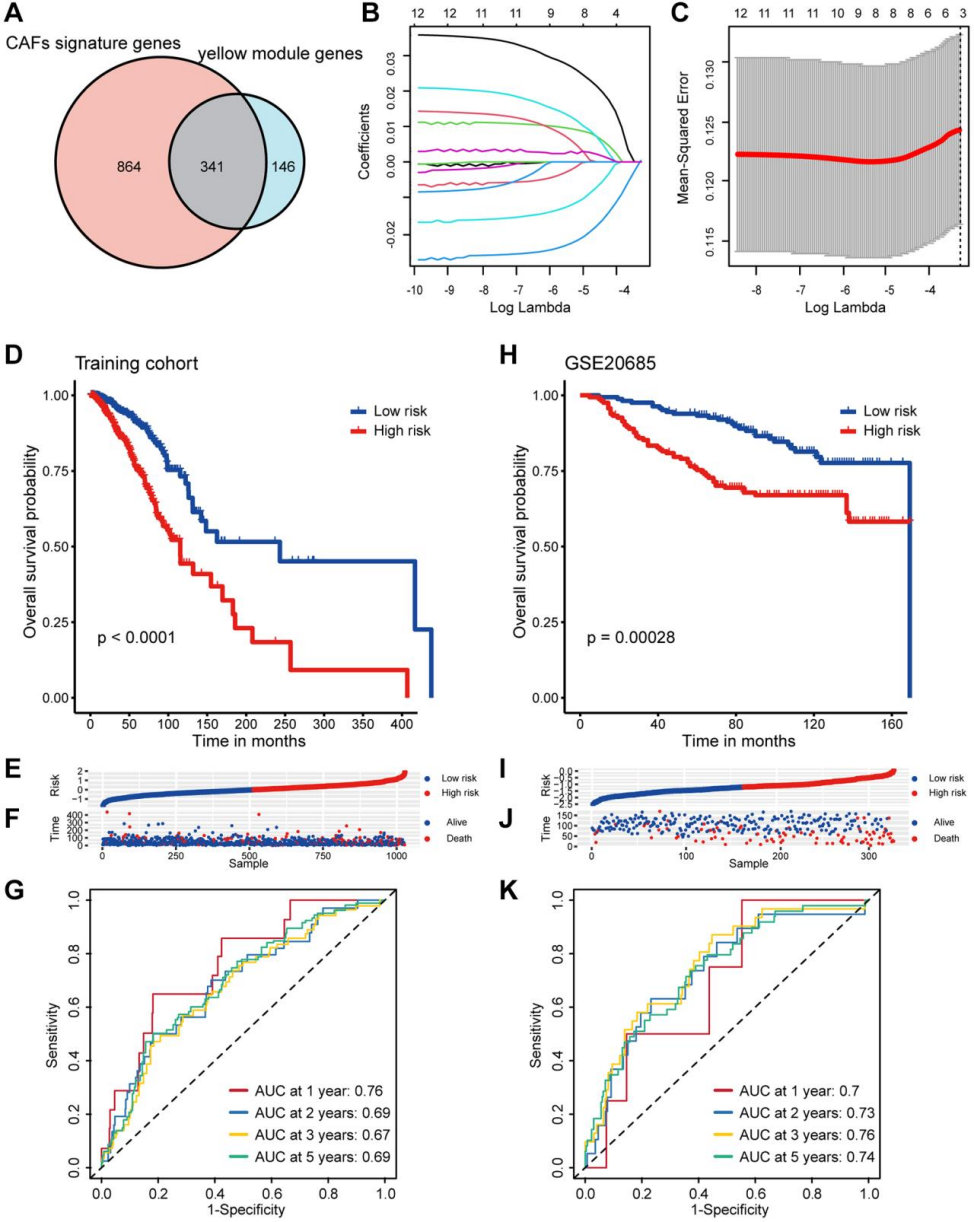
**Screening of CAF-related genes and construction a CAF-related prognostic signature in breast cancer.** (**A**) The Venn graph of the CAF signature genes and yellow module genes. (**B**) Coefficient profiles in the LASSO regression model. (**C**) Cross-validation for tuning parameter selection in the LASSO regression. (**D**) Kaplan-Meier survival analysis was performed on the relationship between the risk score and OS using the TCGA training cohort. (**E**) The rank of risk score in the TCGA training cohort. (**F**) Survival status in the TCGA training cohort. (**G**) Time-dependent ROC curve analysis of the prognostic model (1, 2, 3, and 5 years) in the TCGA training cohort. (**H**) Kaplan-Meier survival analysis was performed on the relationship between the risk score and OS using the GSE20685 validation cohort. (**I**) The rank of risk score in the GSE20685 validation cohort. (**J**) Survival status in the GSE20685 validation cohort. (**K**) Time-dependent ROC curve analysis of the prognostic model (1, 2, 3, and 5 years) in the GSE20685 validation cohort.

By intersecting the 1205 CAFs marker genes with the 487 yellow module genes for fetching, 341 candidate CAF-related genes were obtained to be included in the downstream analysis (Figure 3A). Initially, univariate Cox regression analysis revealed that 12 genes were associated with the prognosis of breast cancer, followed by LASSO analysis to derive a prognostic signature composed of 8 genes (Figure 3B, 3C), including *CERCAM*, *EMP1*, *SDC1*, *PRKG1*, *XG*, *TNN*, *WLS*, and *PDLIM4* (Supplementary Table 3). The TCGA breast cancer cohort was used as the training dataset, and risk score for each sample were computed using the coefficients and expression levels of the prognostic signature genes. We then categorized breast cancer patients into high/low-risk groups in the TCGA training cohort based on median risk score and discovered that patients in the low-risk group had markedly superior outcomes (Figure 3D–3F). Following this, the area under the receiver operating characteristic (ROC) curve (AUC) values for 1, 2, 3, and 5 years were 0.76, 0.69, 0.67, and 0.69 respectively (Figure 3G), which fully demonstrated the excellent performance of our risk model. In order to confirm the reliability and stability of the CAF-related prognostic signature, we validated it in several other additional independent validation datasets. Similarly, consistent with the training set, the breast cancer patients in the validation dataset were stratified based on the median risk score, revealing significantly worse outcomes for patients in the high-risk group (Figure 3H–3J). The AUC values for risk score in the GSE20685 dataset were 0.70 for 1 year, 0.73 for 2 years, 0.76 for 3 years and 0.74 for 5 years respectively (Figure 3K). In addition, breast cancer patients with higher risk score also had significantly shorter survival in the GSE37751 and GSE58812 datasets (Supplementary Figure 2). Overall, we have established and validated an innovative and robust CAF-related prognostic signature for predicting breast cancer prognosis.

### Analyses of clinicopathological characteristics based on the CAF-associated prognostic signature

**Figure 4:**
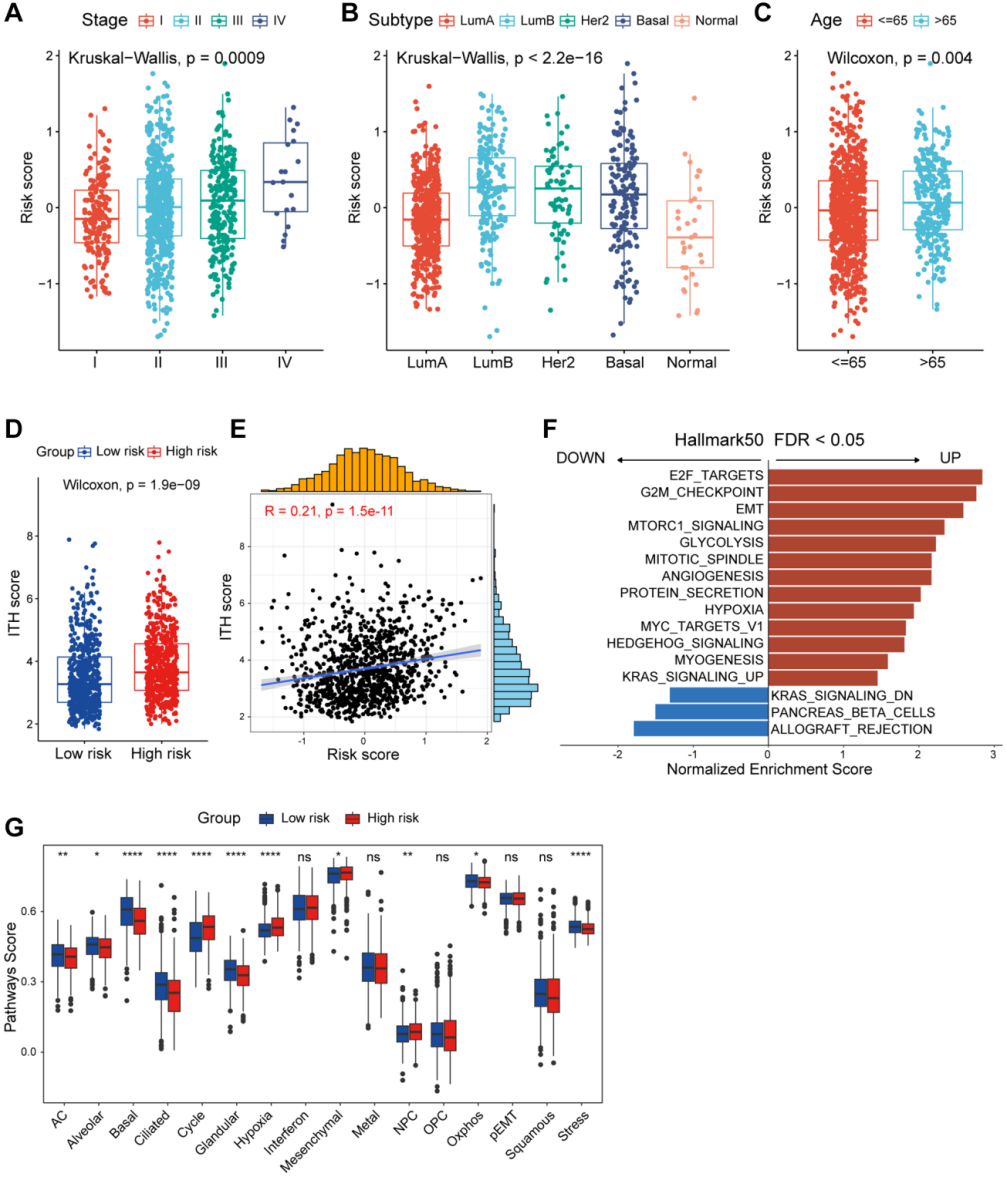
**Functional and genomic features of CAF-related risk score-based classification.** (**A**) Levels of risk score in different tumor stages of breast cancer. (**B**) Levels of risk score in different molecular subtypes of breast cancer. (**C**) Levels of risk score in different age groups of breast cancer. (**D**) Boxplot showing the levels of ITH in high/low-risk groups. Paired two-sided Wilcoxon test. (**E**) Scatter plot showing the correlation between the risk score and ITH score. (**F**) Bar plot showing different pathways enriched in high/low-risk groups of breast cancer calculated by GSEA. (**G**) Boxplots showing the signature score of 16 cancer cell states in high/low-risk groups of breast cancer scored by GSVA. Paired two-sided Wilcoxon test. The asterisks represent the statistical *P*-value (^*^p < 0.05; ^**^p < 0.01; ^***^p < 0.001; ^****^p < 0.0001; ^ns^*p* > 0.05).

Besides the distinct survival outcomes observed between high/low-risk populations, notable variances were also observed in their clinicopathologic features. An incremental rise in the risk score was noted with advancing clinical stages, implying a potential correlation between the risk score and the progression of tumors (Figure 4A). Subsequently, we investigated the prognostic role of risk score in patients within tumor stage I-IV. Patients with lower risk score exhibited significantly better prognoses in stages II and III, while outcomes in stages I and IV did not show a similar improvement (Supplementary Figure 3A–3D). This phenomenon might be attributed to the limited number of patients with stage I and IV breast cancer and the growing emphasis on early breast cancer prevention. Among the different PAM50 molecular subtypes, the risk score exhibited a notable elevation in the LumB subtype and minimal in the normal-like subtype, which accounts for only a small fraction of breast cancers (Figure 4B). Similarly, we investigated the prognostic implications of risk score in patients within different PAM50 subtypes. Analysis revealed that patients with elevated risk score experienced poorer prognose in the Lum A/B and Basal-like subtypes, but not significantly in the Her2 and normal-like subtypes (Supplementary Figure 3E–3I). Furthermore, elderly patients had higher risk score, which may indicate that it played a role in the aging process (Figure 4C). Intra-tumor heterogeneity (ITH) is one of the crucial ingredients contributing to the failure of cancer therapies and patient mortality, higher ITH levels are associated with the development of therapeutic resistance [26]. Conspicuously, patients in high-risk group displayed elevated levels of ITH (Figure 4D). Risk score was positively correlated with ITH score, implying that high-risk patients were more resistant to single treatment (Figure 4E). Summarily, above results showed us high/low-risk breast cancer patients presented with unique clinicopathological profiles.

### Functional analyses of the CAF-related prognostic signature

In order to investigate the underlying mechanisms explaining differences in outcomes and clinical characteristics between risk subgroups, we dissected the differences between subgroups in several ways. Initially, Gene set enrichment analysis (GSEA) analysis (Figure 4F) highlighted that breast cancer patients in the high-risk group showed a considerably enrichment in E2F targets, epithelial-mesenchymal transition (EMT) and angiogenesis (Supplementary Figure 4A–4C). Conversely, allograft rejection, pancreas beta cells and KRAS signaling DN were observed in the low-risk group patients (Supplementary Figure 4D–4F). Barkley D and colleagues innovatively developed multiple gene sets containing 16 recurrent cancer cell states which interacted with the TME to form organizational systems capable of promoting tumor progression, metastasis, and treatment failure [27]. To comprehensively gain insight into transcriptional heterogeneity between high and low risk groups, we calculated 16 recurrent cancer cell states using single sample gene set enrichment analysis (ssGSEA). As depicted in Figure 4G, low-risk patients demonstrated elevated score in astrocyte (AC)-like, alveolar, basic, ciliated, and glandular. Nevertheless, the cycle and hypoxia modules were enriched in high-risk patients. These data may illuminate the potential rationales for the poorer prognosis of high-risk patients.

### Mutational features of the CAF-related prognostic signature

**Figure 5:**
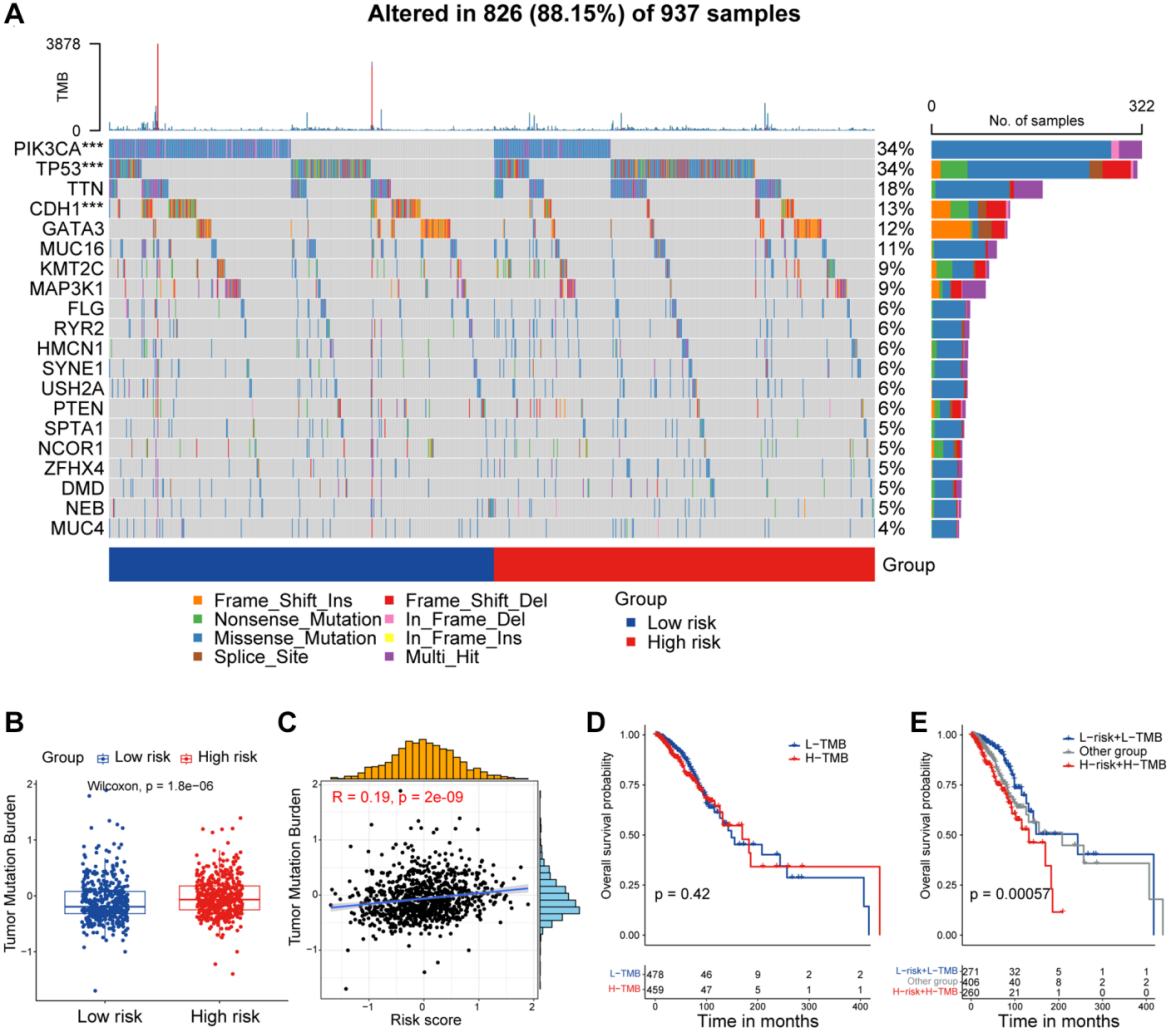
**Mutational feature of the CAF-related prognostic signature.** (**A**) Waterfall plot represents the mutation distribution of the most frequently mutated genes in high/low-risk groups. (**B**) Boxplot showing the levels of TMB in high/low-risk groups. Paired two-sided Wilcoxon test. (**C**) Scatter plot showing the correlation between the risk score and TMB in TCGA cohort. (**D**) Kaplan-Meier survival analysis was performed on the relationship between TMB and OS. (**E**) Kaplan-Meier survival analysis was performed on the relationship between combination of TMB and the risk score and OS. The asterisks represent the statistical *P*-value (^*^p < 0.05; ^**^p < 0.01; ^***^p < 0.001; ^****^p < 0.0001).

One of the most fundamental characteristics of cancer is preternatural and uncontrolled cell growth caused by genome-driven mutations, which in turn affects the homeostatic development of a range of essential cellular functions [28]. We described and compared the mutational features of patients in the high/low-risk groups. Somatic mutations were observed in 88.15% of patients, with a higher frequency of *TP53* mutation, but lower frequency of *PIK3CA* and *CDH1* mutations in the high-risk group (Figure 5A). TMB refers to the aggregate number of substitutions, insertion or deletion mutations per megabase in the exon coding region of a gene in the genome of a tumor cell [29]. Additionally, as an indicator of tumor mutations, TMB is frequently used to predict the efficacy of immunotherapy [30]. Apparently, patients in the high-risk group exhibited higher TMB levels (Figure 5B), with TMB showing a positively correlated with risk score (Figure 5C). Surprisingly, breast cancer patients stratified by the median TMB level did not show significant differences in overall survival expectancies (Figure 5D). Therefore, we investigated how the combination of TMB and CAF-related risk score could jointly categorize breast cancer patients into groups with significantly different prognoses (Figure 5E). To sum up, frequency of somatic mutation was higher in the high-risk group, and integration of CAF-related risk score and TMB could further refine the prediction of prognosis in breast cancer patients.

### Tumor immune microenvironment of the CAF-related prognostic signature

**Figure 6:**
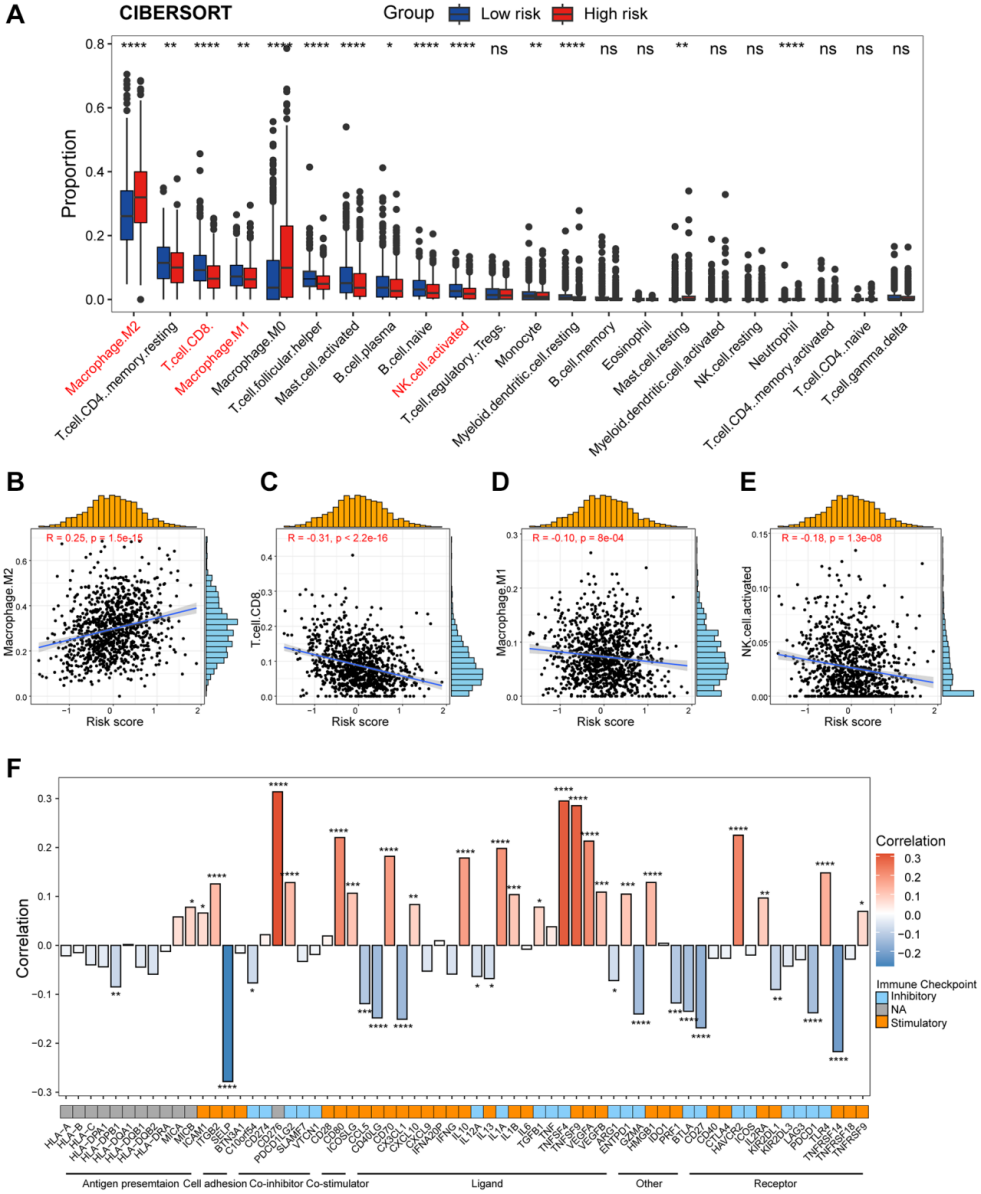
**Dissection of tumor immune microenvironment features between high/low-risk group.** (**A**) Boxplots showing the proportion of 22 immune cells in high/low-risk groups of breast cancer estimated by CIBERSORT. Paired two-sided Wilcoxon test. (**B**–**E**) Scatter plots showing the correlation between the risk score and the proportion of M2 macrophages, CD8^+^ T cells, M1 macrophages, and activated NK cells. (**F**) Bar plot of the correlation between immunomodulators and the risk score in TCGA cohort. The asterisks represent the statistical *P*-value (^*^p < 0.05; ^**^p < 0.01; ^***^p < 0.001; ^****^p < 0.0001; ^ns^*p* > 0.05).

In this section, we aimed to investigate the disparities in the tumor immune microenvironment (TIME) between the two risk groups. First, we assessed the distribution of immunocyte percentage in each patient by the CIBERSORT algorithm (Figure 6A). Apparently, M2 macrophages were more prevalent in high-risk patients, and correlation analysis showed a significant positive correlation between the percentage of M2 macrophages and the risk score (Figure 6B). In addition, CD8^+^ T cells, M1 macrophages, and activated NK cells were more enriched in patients in the low-risk group, with their infiltrations were negatively correlated with risk score (Figure 6C–6E). We also calculated immune cell infiltration utilizing additional algorithms, including CIBERSORT. ABS and MCPCOUNTER, and rightfully so these results are consistent with CIBERSORT (Supplementary Figure 5). Subsequently, we collected 65 immunomodulators from various sources in the literature, and as depicted in Figure 6F we calculated the correlation between these immunomodulators and the CAF-associated risk score. In particular, risk score was significantly positively correlated with the immunosuppressive molecules *CD276*, *TNFSF9*, and *HAVCR2*, and significantly negatively correlated with the stimulatory immune checkpoint markers *SLEP* and *TNFRSF14*.

**Figure 7:**
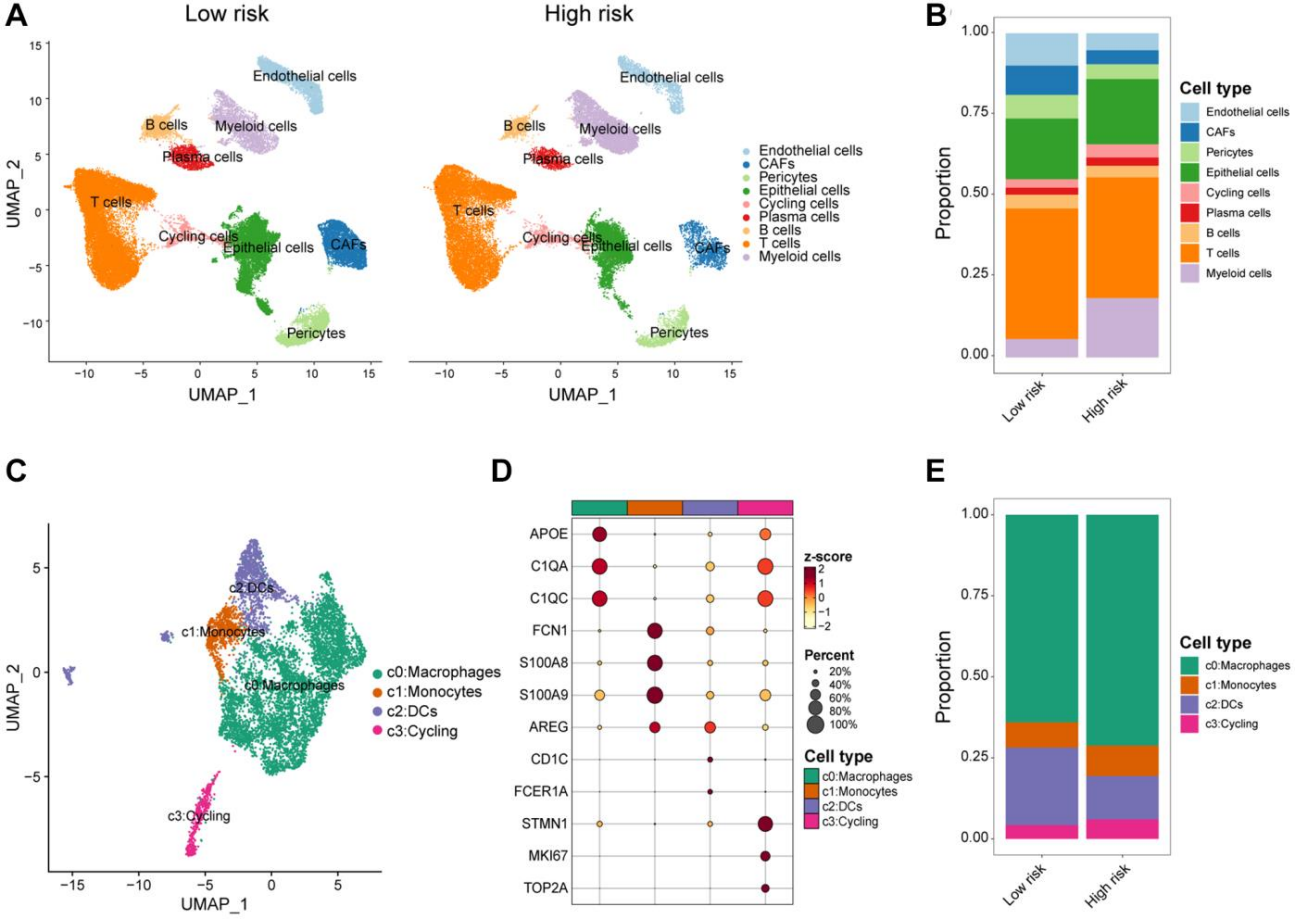
**scRNA-seq analysis of the tumor immune microenvironment features between high/low-risk group.** (**A**) UMAP plot showing the major cell subpopulations of high- and low-risk breast tumors. (**B**) Relative proportions of diverse cell types across high/low-risk tumors. (**C**) UMAP plot showing the diverse subsets of myeloid cells in breast cancers. (**D**) Bubble heatmap showing expression levels of selected signature genes for myeloid cells in breast cancers. Dot size indicates fraction of expressing cells, colored based on normalized expression levels. (**E**) Relative proportions of diverse subpopulations of myeloid cells across high/low-risk tumors.

In addition, we capitalized on the previously mentioned scRNA-seq data which contains 24 paired breast cancer bulk RNA-seq data. According to the median risk score calculated by bulk RNA-seq data, these 24 samples were stratified into high/low-risk group (Figure 7A). Patients in High/low-risk group displayed contrasting cellular subpopulation compositions (Figure 7B). Notably, myeloid cells, known for typically exerted an immunosuppressive effect, were more abundant in high-risk samples (Figure 7B). This finding corresponded with our CIBERSORT classification of immune cell infiltration analysis described above. To comprehensively gain an understanding of myeloid cells, we further re-clustered myeloid cells and distinguished macrophages, monocytes, dendritic cells (DCs) and cycling cells based on multiple cellular markers (Figure 7C, 7D). The results showed us that patients in the high-risk group had significantly higher composition of macrophages, which predominantly contribute to suppressing anti-cancer immunity (Figure 7E). Overall, these data illustrated distinct differences in the immune features of the two groups, with patients in the high-risk group demonstrating an immunosuppressive phenotype.

### Correlation between anti-cancer drug sensitivities and the CAF-related prognostic signature score

**Figure 8:**
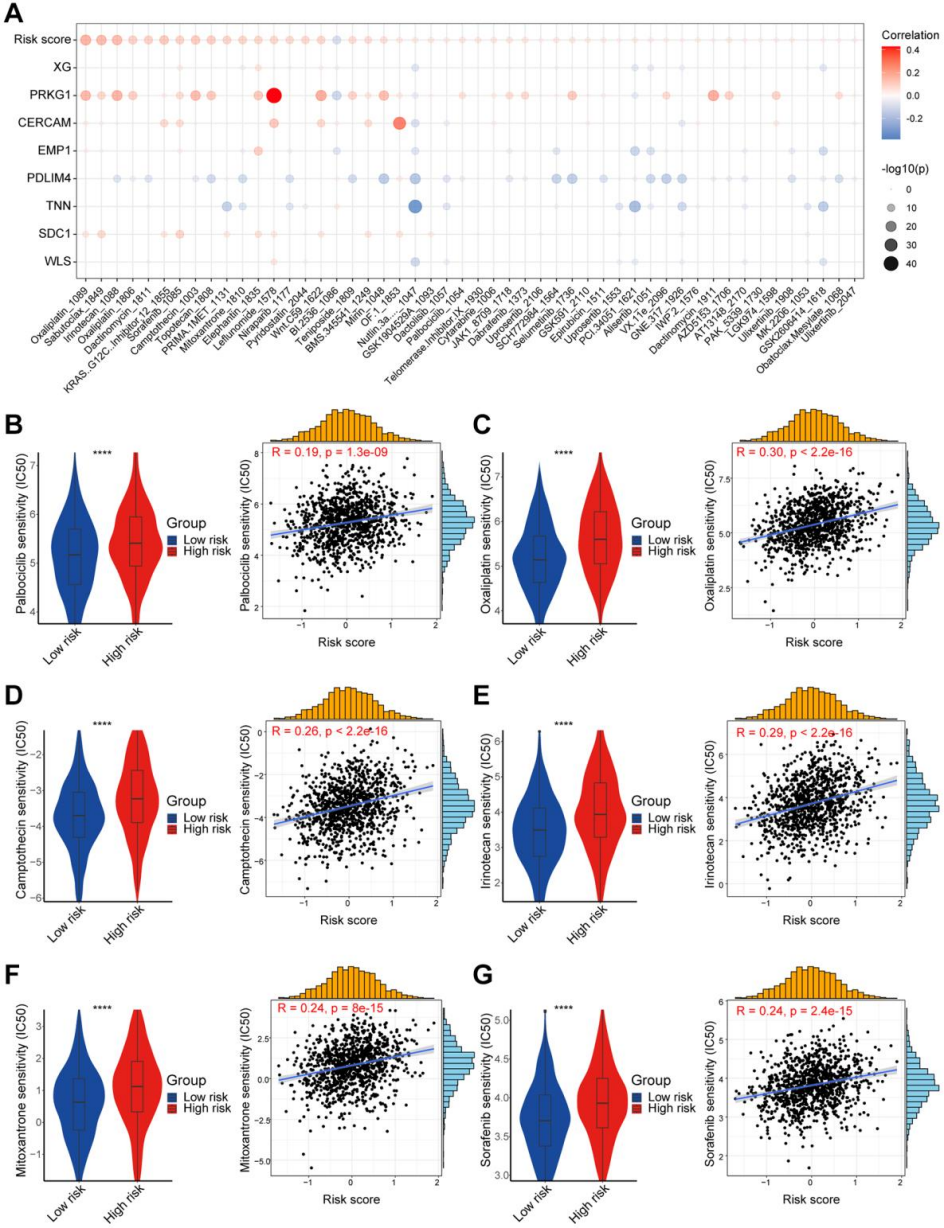
**High- and low-risk group patients differ in drug sensitivity.** (**A**) Bubble plot showing the relationship between IC50 of drugs, risk score, and model genes. (**B**) Boxplot showing the comparison of IC50 of Palbociclib between high/low-risk groups, and scatter plot showing the correlation between the IC50 of drug and risk score. (**C**) Boxplot showing the comparison of IC50 of Oxaliplatin between high/low-risk groups, and scatter plot showing the correlation between the IC50 of drug and risk score. (**D**) Boxplot showing the comparison of IC50 of Camptothecin between high/low-risk groups, and scatter plot showing the correlation between the IC50 of drug and risk score. (**E**) Boxplot showing the comparison of IC50 of Irinotecan between high/low-risk groups, and scatter plot showing the correlation between the IC50 of drug and risk score. (**F**) Boxplot showing the comparison of IC50 of Mitoxantrone between high/low-risk groups, and scatter plot showing the correlation between the IC50 of drug and risk score. (**G**) Boxplot showing the comparison of IC50 of Sorafenib between high/low-risk groups, and scatter plot showing the correlation between the IC50 of drug and risk score. The asterisks represent the statistical *P*-value (^****^p < 0.0001).

In order to delve deeper into the clinical implications of risk score and prognostic genes, we calculated the half-maximal inhibitory concentration (IC50) values for various drugs in breast cancer patients in TCGA using the GDSC database. Figure 8A showed the correlation between drug sensitivities and the risk score as well as prognostic genes. A notable positive correlation was observed between the risk score and IC50 of Oxaliplatin, Sabutoclax, and Irinotecan. However, the IC50 of BI.2536, a potent highly selective *PLK1* inhibitor, negatively correlated with the CAF-associated risk score. Interestingly, the prognostic gene *PRKG1* was highly correlated with the IC50 of Leflunomide (Supplementary Figure 6A), and *TNN* was negatively correlated with the IC50 of Nutlin.3a .... _1047, a potent *MDM2* inhibitor (Supplementary Figure 6B). Additionally, we compared the IC50 levels of a series of Food and Drug Administration (FDA)-approved breast cancer treatments, and the IC50 levels were higher in the high-risk group for Palbociclib, Oxaliplatin, Camptothecin, Irinotecan, Mitoxantrone, and Sorafenib. These results suggested that breast cancer patients with lower risk score might be sensitive to these FDA-approved drugs (Figure 8B–8G). The I-SPY2 trial platform is a continuous, multicenter, Phase II neoadjuvant trial platform for high-risk, early-stage breast cancer [31]. We calculated the CAF-related risk score for each patient and found higher risk score for pathological complete response (pCR) patients in the Pertuzumab (Paclitaxel + Pertuzumab + Trastuzumab) and TMD1/P (T-DM1 + Pertuzumab) subgroups of the 10 cancer treatment arms for which they were advertised (Supplementary Figure 6C). In the GSE123845 dataset, which was breast cancer data after standard neoadjuvant therapy, patients with higher risk score demonstrated a favorable prognosis (Supplementary Figure 6D). In summary, the above findings defined that the CAF-related risk score could serve as a reputable tool for predicting drug sensitivity and response to cancer therapy for breast cancer patients.

### Constructing a nomogram of the CAF-related prognostic signature

**Figure 9:**
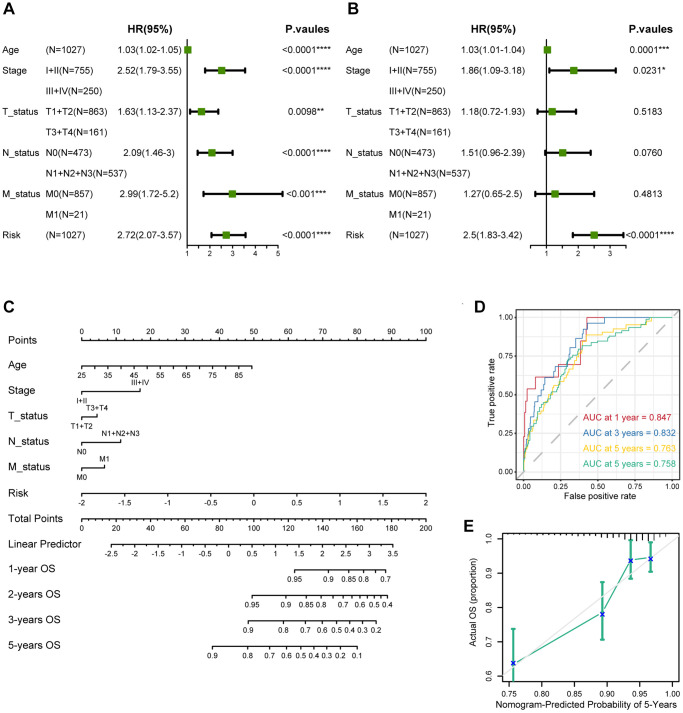
**Establishment and assessment of the nomogram survival model.** (**A**) Univariate analysis for the clinicopathologic characteristics and the risk score in TCGA cohort. (**B**) Multivariate analysis for the clinicopathologic characteristics and the risk score in TCGA cohort. (**C**) A nomogram was established to predict the prognosis of breast cancer patients. (**D**) Time-dependent ROC curve analysis of the nomogram (1, 2, 3, and 5 years) in TCGA cohort. (**E**) Calibration plots showing the probability in TCGA cohort. The asterisks represent the statistical *P*-value (^*^p < 0.05; ^**^p < 0.01; ^***^p < 0.001; ^****^p < 0.0001).

Both univariate and multifactorial Cox analyses revealed that age, tumor stage, and CAF-related risk score were independent prognostic factors in breast cancer patients (Figure 9A, 9B). Therefore, a nomogram was developed incorporating the CAF-related risk score with additional clinical information on other independent predictors proposed through multivariate Cox analysis. In the nomogram, CAF-related risk score contributed significantly to the prediction of survival probability, serving as a quantitative and visual tool for predicting 1-, 3-, and 5-year OS (Figure 9C). The AUCs for 1-, 2-, 3-, and 5-year OS in the nomogram were 0.847, 0.832, 0.763, and 0.758, respectively, suggesting a significantly better prognostic ability than the CAF-related risk score alone (Figure 9D). In addition, calibration curves were presented to assess the performance of the nomograms, which showed that the predicted curves of the model are close to the ideal ones (Figure 9E). These results highlighted that significant predictive impact of the nomogram model on breast cancer patients.

## DISCUSSION

Breast cancer, the gravest neoplastic affliction affecting women worldwide, is a multifaceted and exceedingly heterogeneous ailment [32]. Tumor heterogeneity refers to the variance in patients’ response to identical treatments [33]. The tumor microenvironment (TME) encompasses non-malignant cells and constituents within the tumor, as well as the molecules they generate and emit. It is widely recognized that the direct and indirect interactions between tumor cells and the TME are pivotal in the genesis, progression, metastasis, and therapy of the malignancy. CAFs occupy a central role in the TME, exercising indispensable roles in extracellular matrix remodeling, maintenance of stem cell characteristics, angiogenesis, regulation of tumor metabolism, immune response modulation, and facilitation of cancer cell proliferation, migration, invasion, and resistance to therapeutic interventions through manifold mechanisms. Literature reports outline that CAFs are capable of establishing interactive associations with other cells in TME, thereby establishing an immunosuppressive loop that further enhances immune inhibition in the TME. For instance, in triple-negative breast cancer (TNBC), CAFs lend support to immunosuppression by recruiting monocytes to the tumor via the CXCL12-CXCR4 axis, thereby inducing their transformation into tumor-promoting lipid-associated macrophages [34]. Furthermore, CAFs have been scientifically demonstrated to impede CD8^+^ T cell infiltration and bestow resistance against immune checkpoint blockades (ICBs) therapies [35]. The firmly established capability of CAFs to promote tumorigenesis designates them as promising targets for immunotherapeutic interventions [36]. Nonetheless, precise biomarkers specifically linked with CAFs in breast cancer remain ambiguous, presenting significant challenges in clinical management. We are steadfast in our resolve to explore novel markers associated with CAFs in breast cancer and contribute towards improving clinical treatment strategies.

Bulk RNA-seq examines variations between tissue samples at the genomic, transcriptomic, and epigenomic levels. In contrast, single-cell RNA-seq is a sequencing analysis technique performed at the individual cell level, elucidating differences within tissue samples [37]. The combination of these two methodologies offers a comprehensive, multi-omics investigation into the heterogeneity of tumor tissue. The integration of scRNA-seq and bulk RNA-seq carries significant potential in revealing profound biological insights and represents the prevailing trend and future trajectory of histological analysis. In this study, we re-analyzed single cell sequencing data with 26 samples breast cancer and distinctly identified fibroblast subpopulations, defining 1,205 CAFs markers. To explore biological markers of CAFs in bulk RNA-seq, we performed WGCNA. To ensure the robustness of the marker genes, we employed three bioinformatics methodologies to quantify the infiltration of CAFs in breast cancer tissue. Module correlation analysis revealed a robust association between the yellow module and all three methods, thereby establishing the yellow module as a marker for CAFs in bulk RNA-seq. The marker genes derived from scRNA-seq were intersected with the CAFs module genes in bulk RNA-seq, resulting in the identification of 341 genes pertaining to CAFs. Subsequently, univariate Cox and LASSO regression analyses were carried out to screen for eight candidate genes with prognostic relevance, comprising five risk genes (*CERCAM*, *EMP1*, *SDC1*, *PRKG1*, *XG*) and three protective genes (*TNN*, *WLS*, *PDLIM4*). While some of these eight candidate genes have already been investigated for their noteworthy roles in tumors, including breast cancer, others await more comprehensive examination. For instance, *EMP1* has been recognized as a biomarker for gefitinib resistance in lung cancer and contributes to prednisolone resistance in patients with acute lymphoblastic leukemia [38]. *SDC1* functions as a cell surface proteoglycan to attach the cytoskeleton to the mesenchymal matrix and regulates exosome biogenesis. Furthermore, *SDC1* can promote the progression of triple-negative breast cancer by activating the c-src/FAK signaling pathway [39]. We consider *SDC1* to be a focal point for our future research endeavors. Grounded on these eight prognostically relevant genes, we developed a CAF-related prognostic model and calculated a CAF-related risk score for each breast cancer patient. Based on the median risk score, patients were categorized into high/low-risk groups, with individuals in the high-risk category exhibiting a notably poorer prognosis. Furthermore, to validate the stability of this prognostic model, we subjected it to verification using three independent validation sets. Intriguingly, the risk score exhibited a significant correlation with clinical stage, PAM50 staging, and age of the patients. Moreover, patients with higher risk score also displayed increased ITH, cancer gene mutations, and activation of pro-cancer molecular pathways. Importantly, patients with lower risk score demonstrated significantly higher infiltration of immune cells, including CD8^+^ T cells, M1 macrophages, and NK cells, whereas high-risk patients showed elevated M2 macrophage infiltrates. Additionally, we delved into the TIME at the single-cell level and noted increased infiltration of myeloid cells, specifically macrophages and monocytes, in high-risk patients, alongside decreased infiltration of DCs. Consequently, the elimination of M2 macrophages or their progenitors may serve as a promising therapeutic strategy for patients in high-risk populations. We further screened breast cancer patients in the high/low-risk groups for a variety of drugs and identified Palbociclib, Oxaliplatin, and Camptothecin as especially suitable options for individuals in the low-risk category. Most notably, we developed an effective prognostic marker based on CAF-related genes and presented a novel and accurate classification system, alongside therapeutic strategies, for breast cancer patients.

Currently, the management of breast cancer predominantly still rely on clinical staging and pathologic staging [40]. In this study, we observed a progressive increase in the risk score corresponding to the clinical stage, with patients in stages III and IV displaying significantly higher risk score compared to patients in stages I and II. Furthermore, disparities in risk score across patients classified under different PAM50 subtypes, with higher score observed in LumB and Her2 patients, and the lowest score in normal-like patients. Interestingly, we found that risk score tended to be higher in breast cancer patients aged 65 and above, indicating a potential association with the aging process. Given the inherent heterogeneity of tumors, especially in breast cancer, it becomes crucial to investigate the underlying mechanisms and transcriptional differences that contribute to divergent survival outcomes between high and low-risk patient groups. ITH represents a source of genetic variation that can promote cancer progression and the development of drug resistance, consequently culminating in treatment failure. Higher levels of ITH indicate an increased propensity of acquiring drug resistance. Our findings revealed a markedly higher ITH score among high-risk patients, with a positive correlation between risk score and ITH score. The increased heterogeneity and resistance to treatment may contribute to the unfavorable prognosis observed in high-risk patients. Through GSEA enrichment analysis, we identified enrichment of E2F targets, G2M checkpoint, and MITOTIC spindle pathways in the high-risk group, pathways typically associated with cell cycle regulation [41]. This implies a disruption in the regulation of cell cycle progression within high-risk group. Additionally, we observed elevated score for EMT, glycolysis, angiogenesis, and hypoxia in the high-risk group, which aligns with well-established knowledge that CAFs can secrete VEGF to regulate tumor vascular networks, induce EMT in epithelial cells, and contribute to metabolic reprogramming in tumor cells [42]. Our findings further corroborate these associations. The observed heterogeneity in cancer cells can largely be attributed to the redeployment of modules typically expressed in different cellular and developmental contexts. In our investigation, we scrutinized unique attributes of two risk categories by elucidating recently defined cancer cell states that represent fundamental units of tumor transcriptional variability. Notably, the high-risk group exhibited higher characterization score for cycle and hypoxia, while displaying lower score for astrocyte (AC)-like, alveolar, basic, ciliated, and glandular states. TMB not only reflects the extent of genetic alterations within tumor but also provides insight into its immunogenicity. TMB has been identified as a predictive marker for ICB response in melanoma, reflecting its association with immunogenicity in this context [43]. Our study demonstrated elevated TMB in the high-risk group, positively correlating with the risk score. Furthermore, the combined assessment of TMB and CAF-related risk score exhibited strong prognostic performance in predicting patient survival.

While previous research has emphasized the role of transcriptional and epigenetic variation within tumor cells in tumor development, recent attention has transitioned towards the TIME, which encompasses tumor cells, stromal cells, and infiltrating immune cells. Distinct immune microenvironments can influence the response of tumor cells to both the host’s immune system and external treatments, playing a crucial role in tumor immune evasion and drug resistance [44]. Macrophages typically function in phagocytosis and removal of cellular debris, whereas under specific circumstances they polarize into classically activated M1 macrophages and alternatively activated M2 macrophages [45, 46]. Polarization of M1-polarized macrophages can be stimulated by Th1 cytokines such as interferon-gamma (IFN-γ), tumor necrosis factor-α (TNF-α), and bacterial lipopolysaccharide (LPS), whereas M2-polarized macrophages can be triggered by interleukin 4 (IL-4), interleukin 13 (IL-13), and transforming growth factor-β (TGF-β) [47, 48]. Classically activated M1 macrophages have potent anti-infective, anti-tumor, and removal of apoptotic cells and necrotic tissues, whereas selectively activated M2 macrophages are mainly involved in their healing, angiogenesis, and immunosuppression [49]. Within the TIME, macrophages can be induced by CAFs to differentiate predominantly into M2 macrophages, which are known to promote tumor growth and metastasis. Furthermore, CAFs possess the ability to attract infiltration and aggregation of M2 macrophages through the secretion of various growth factors and cytokines. Our findings revealed significantly elevated levels of M2 macrophage infiltration, accompanied by decreased levels of M1 macrophages, in patients classified as high-risk. Furthermore, both CD8^+^ T cells and activated NK cells, which are critical subsets of the immune system with anti-tumor effects, displayed reduced infiltration levels in the high-risk group. Additionally, our analysis at the single-cell level demonstrated that the high-risk population exhibited higher levels of macrophage infiltration and lower levels of DCs. Consequently, the elimination of M2 macrophages or their progenitors presents itself as a potential therapeutic strategy for individuals within high-risk populations.

After exploring the differences between the transcriptional, genetic, and immune microenvironments between high/low-risk risk groups, we worked to screen several anticancer medications for different breast cancer patients. The IC50 of drugs such as Palbociclib, Oxaliplatin, and Camptothecin was significantly lower in the low-risk group. Refreshingly, in the I-SPY2 data, patients with non-responding Her2-positive metastatic breast cancer treated with patuximab had lower risk score. It has been shown that PDPN-positive CAF can promote resistance to trastuzumab in Her2-positive breast cancer by secreting the immunosuppressive factors indoleamine 2,3-dioxygenase 1 (IDO1) as well as tryptophan 2,3-dioxygenase 2 (TDO2) [50]. This could potentially provide a framework for guiding the treatment of breast cancer patients. The nomogram enables integration of our model with standard clinical variables of the patient, including age, pathological staging, etc., thereby offering clinical guidance for personalized treatment. However, the exact function of the above treatments remains to be further confirmed in future prospective studies.

While our prognostic model for breast cancer, based on cancer-associated fibroblasts, demonstrated commendable performance in both the training and validation cohorts, it is imperative to acknowledge certain constraints. Primarily, it is essential to substantiate the expression and prognostic significance of marker genes associated with candidate CAFs at the protein level through further examination. Moreover, despite screening these prognostic genes based on CAFs markers, it should be noted that they are not exclusively expressed by CAFs. Lastly, we must be mindful of the potential presence of inherent bias resulting from the retrospective recruitment of breast cancer patients.

## Supplementary Materials

Supplementary Figures

Supplementary Tables

Supplementary Table 3
