# Supplementary material for: Cancer-associated fibroblast-derived gene signature discriminates distinct prognoses by integrated single-cell and bulk RNA-seq analyses in breast cancer
Source: Aging (Albany NY). 2024 May 9;16(9):8279–305. doi: 10.18632/aging.205817 (PMC11132004; doi:10.18632/aging.205817)
Supplement: Supplementary Figures [file aging-16-205817-s001.pdf]

SUPPLEMENTARY FIGURES

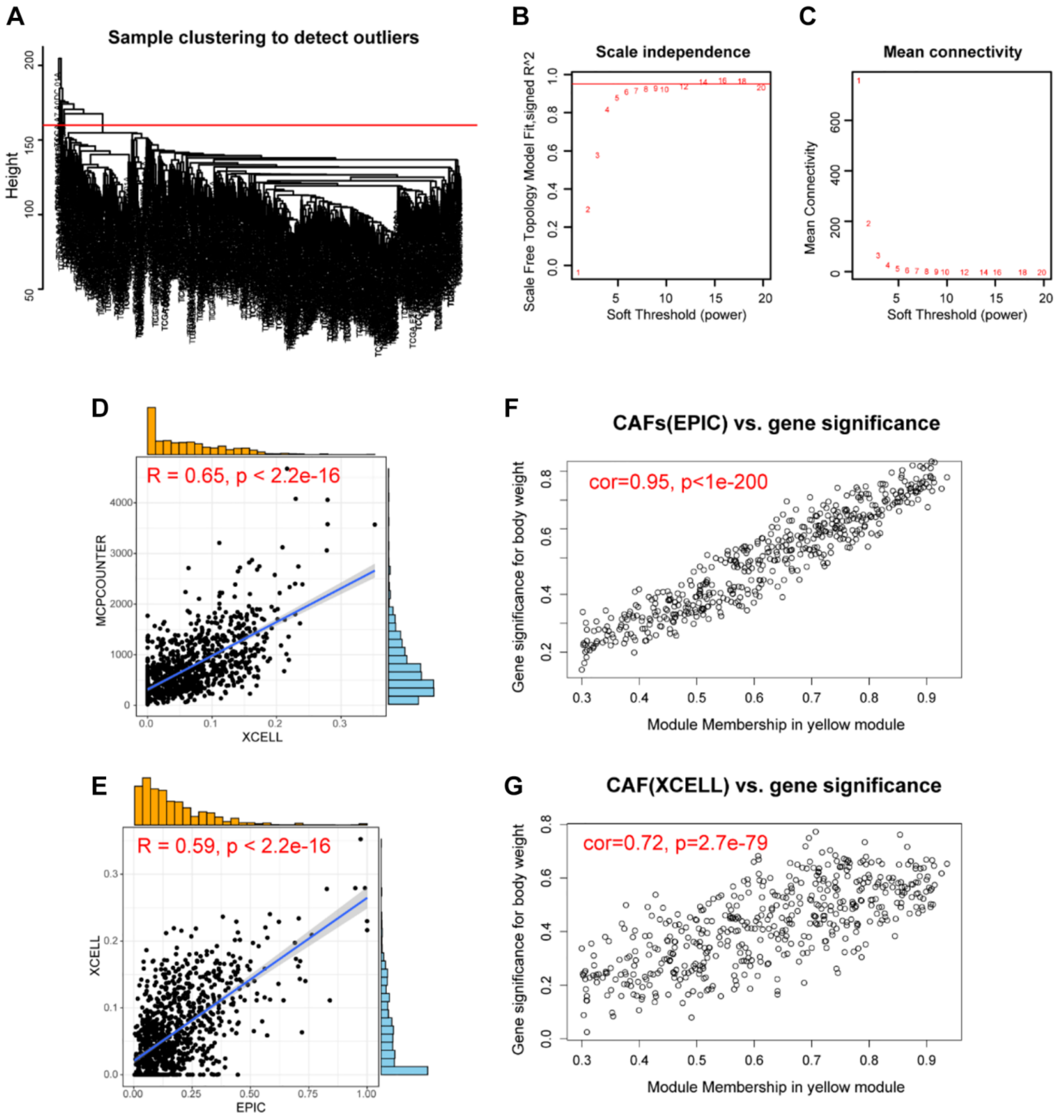

Supplementary Figure 1. Screening for CAF-related genes by WGCNA in breast cancer. (**A**) Samples were clustered and outlier samples were not found. (**B**, **C**) According to the instructions of the WGCNA package, 5 was selected as the soft threshold power. (**D**) Correlation plot of infiltration of CAFs by XCELL and MCPCOUNTER. (**E**) Correlation plot of infiltration of CAFs by EPIC and XCELL. (**F**) Scatter plot between the yellow module and EPIC. (**G**) Scatter plot between the yellow module and XCELL.

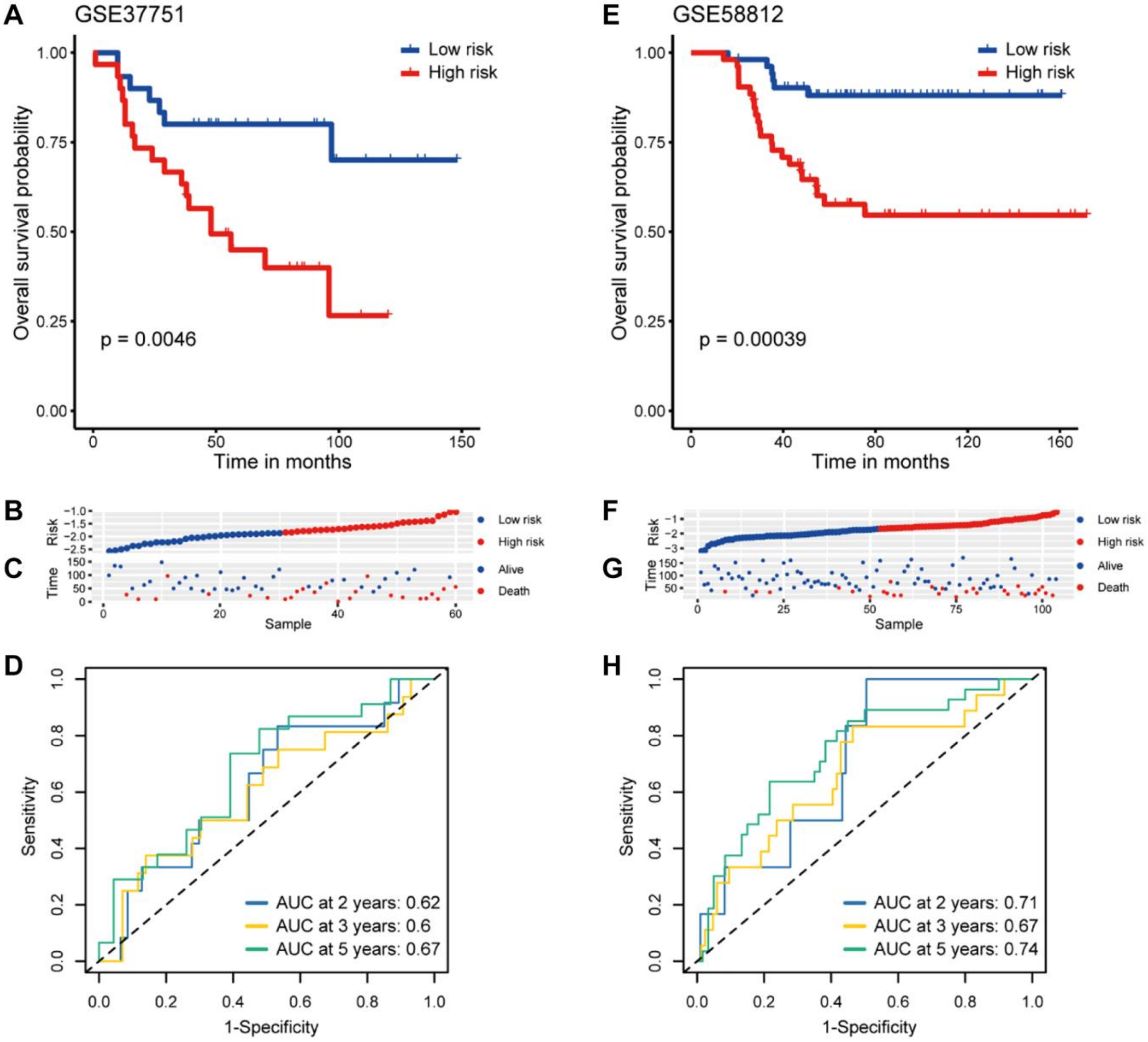

Supplementary Figure 2. Validation of the CAF-related prognostic signature. (**A**) Kaplan-Meier survival analysis was performed on the relationship between the risk score and OS using the GSE37751 validation cohort. (**B**) The rank of risk score in the GSE37751 validation cohort. (**C**) Survival status in the GSE37751 validation cohort. (**D**) Time-dependent ROC curve analysis of the prognostic model (2, 3, and 5 years) in the GSE37751 validation cohort. (**E**) Kaplan-Meier survival analysis was performed on the relationship between the risk score and OS using the GSE58812 validation cohort. (**F**) The rank of risk score in the GSE58812 validation cohort. (**G**) Survival status in the GSE58812 validation cohort. (**H**) Time-dependent ROC curve analysis of the prognostic model (2, 3, and 5 years) in the GSE58812 validation cohort.

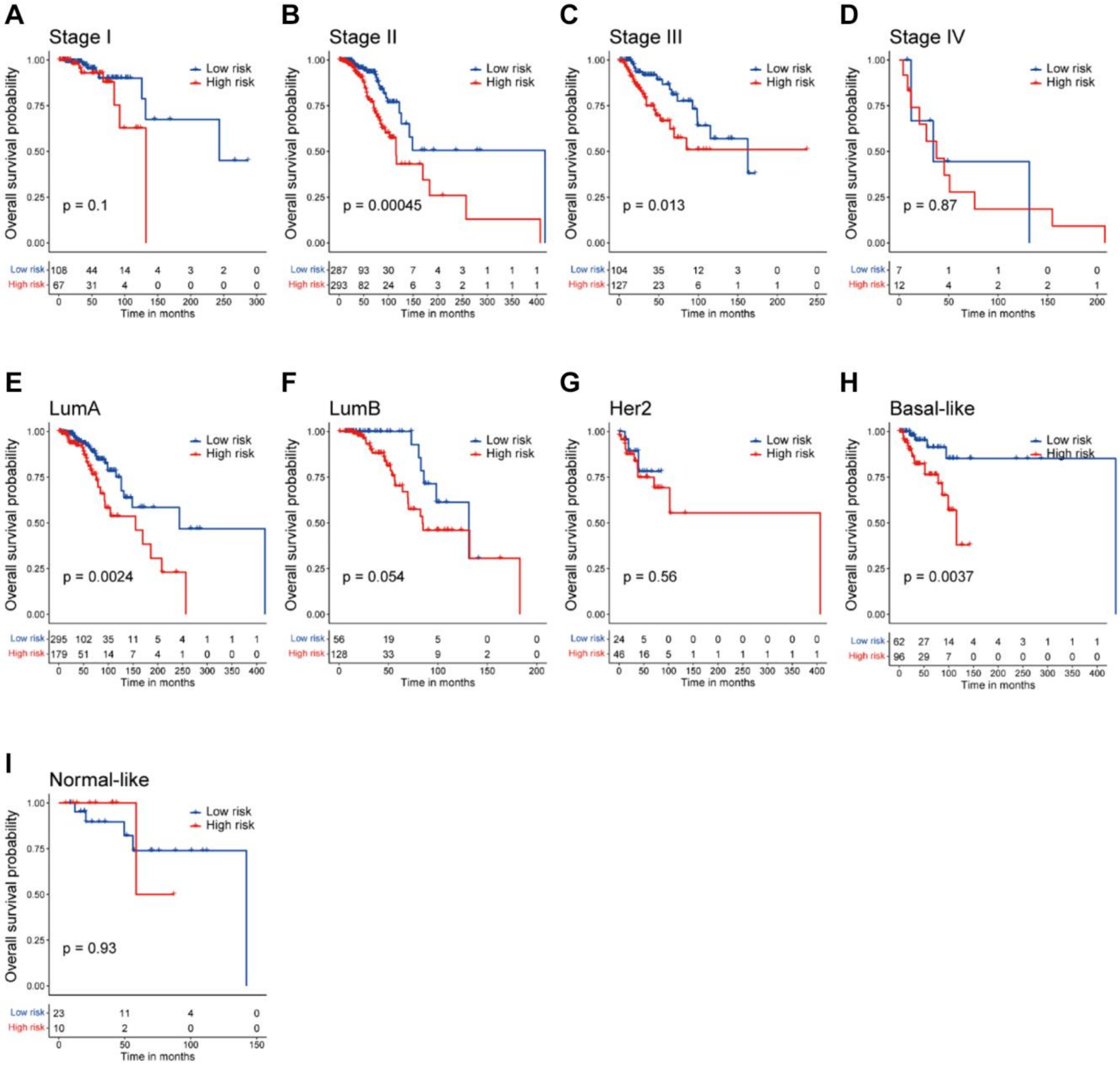

Supplementary Figure 3. Correlation between clinical characteristics and the risk score in breast cancer. (**A**–**D**) Kaplan-Meier survival analyses were performed on the relationship between the risk score and OS in the STAGE I, II, III and IV of breast cancer. (**E**–**I**) Kaplan-Meier survival analyses were performed on the relationship between the risk score and OS in the luminal A/B, HER2-enriched, basal-like and normal-like subtypes of breast cancer.

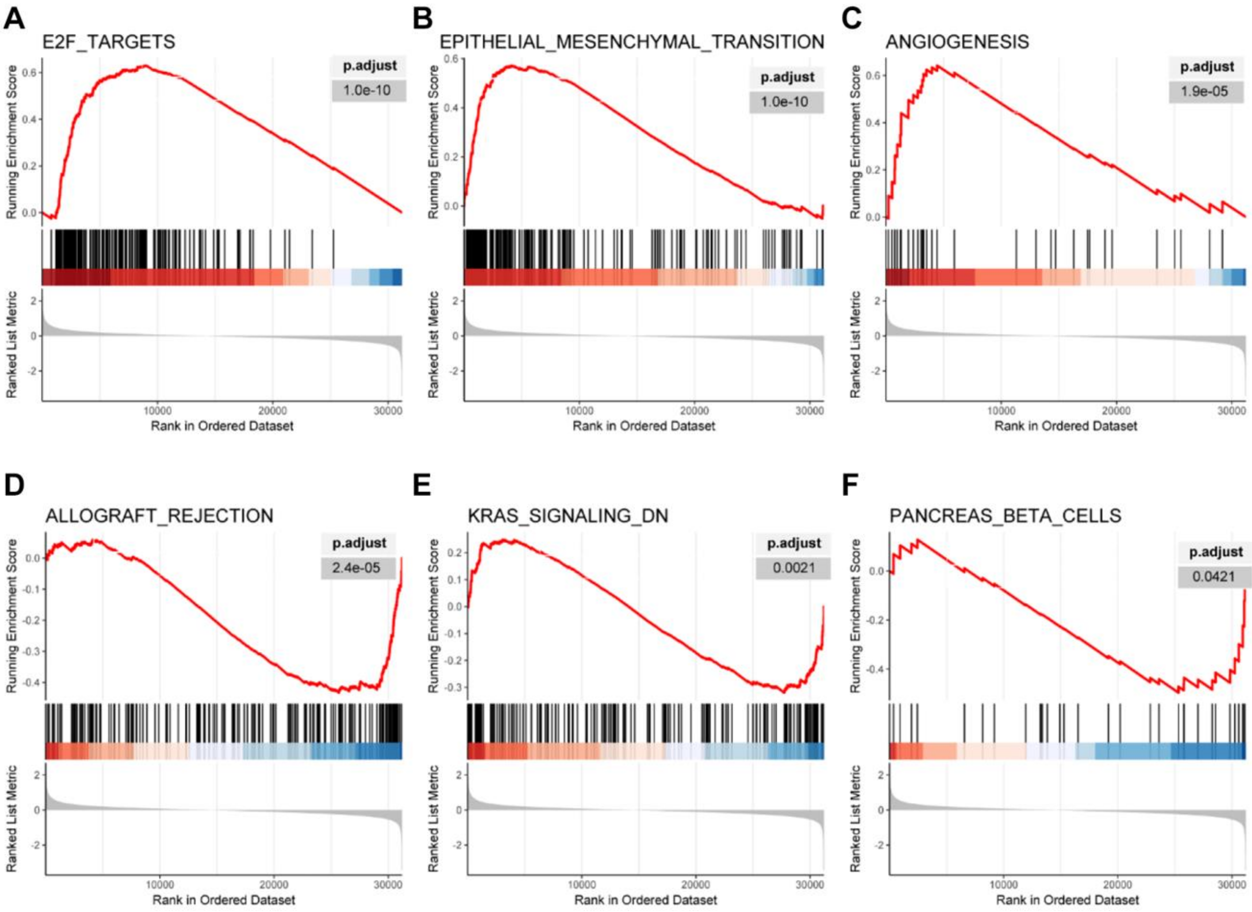

Supplementary Figure 4. Correlation between the risk score and pathway activities. (**A**–**C**) GSEA analysis showing the up-regulated pathways in the high-risk group. (**D**–**F**) GSEA analysis showing the up-regulated pathways in the low-risk group.

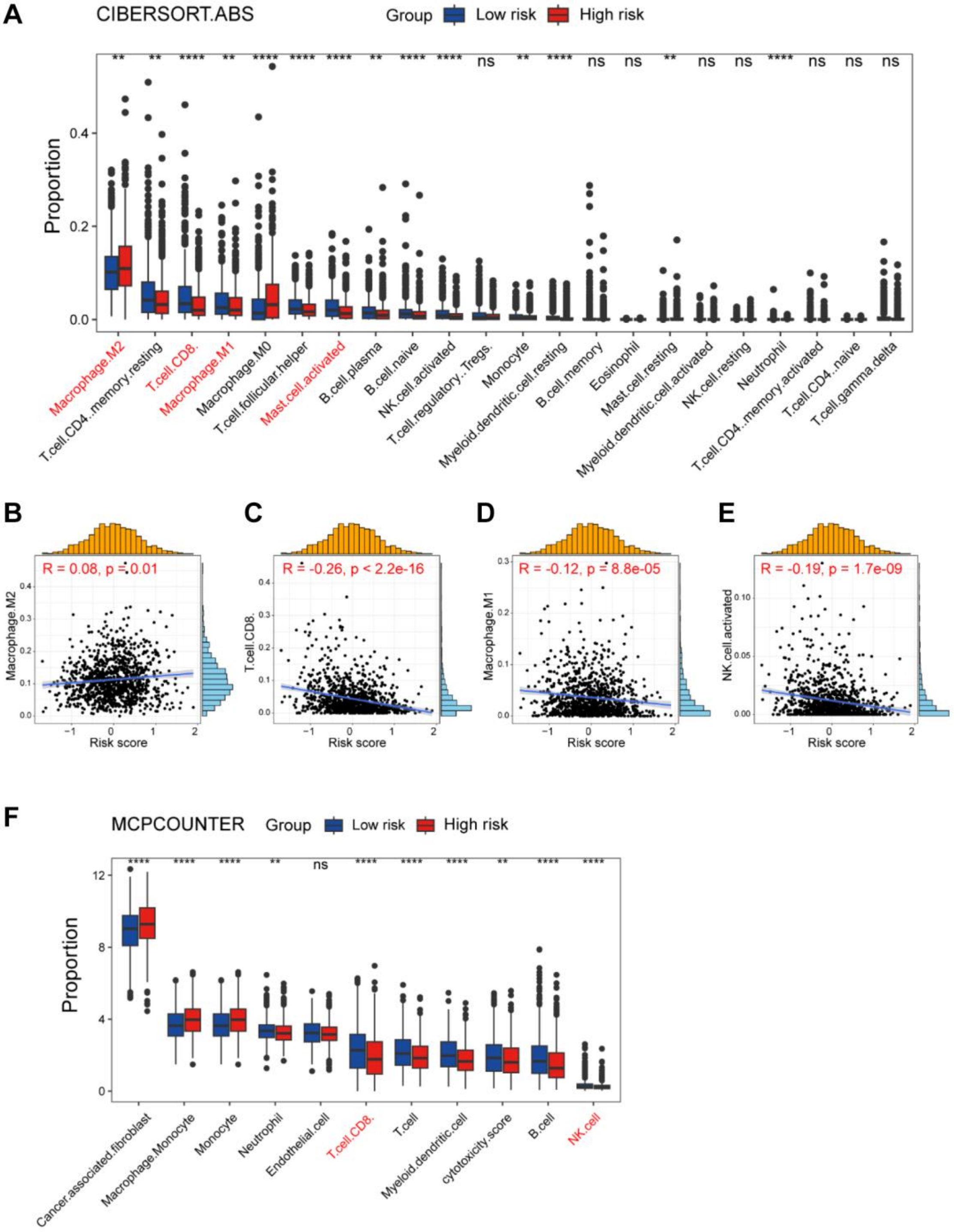

Supplementary Figure 5. Dissection of tumor immune microenvironment features based on the CAF-related prognostic signature. (**A**) Boxplots showing the proportion of 22 immune cells in high/low-risk groups of breast cancer estimated by CIBERSORT.ABS. Paired two-sided Wilcoxon test. (**B**–**E**) Scatter plots showing the correlation between the risk score and the proportion of M2-like macrophages, CD8+ T cells, M1-like macrophages and activated NK cells. (**F**) Boxplots showing the proportion of 22 immune cells in high/low-risk groups of breast cancer estimated by MCPCOUNTER. Paired two-sided Wilcoxon test. The asterisks represent the statistical *P*-value ($^{*}p < 0.05$; $^{**}p < 0.01$; $^{***}p < .001$; $^{****}p < 0.0001$; $^{ns}p > 0.05$).

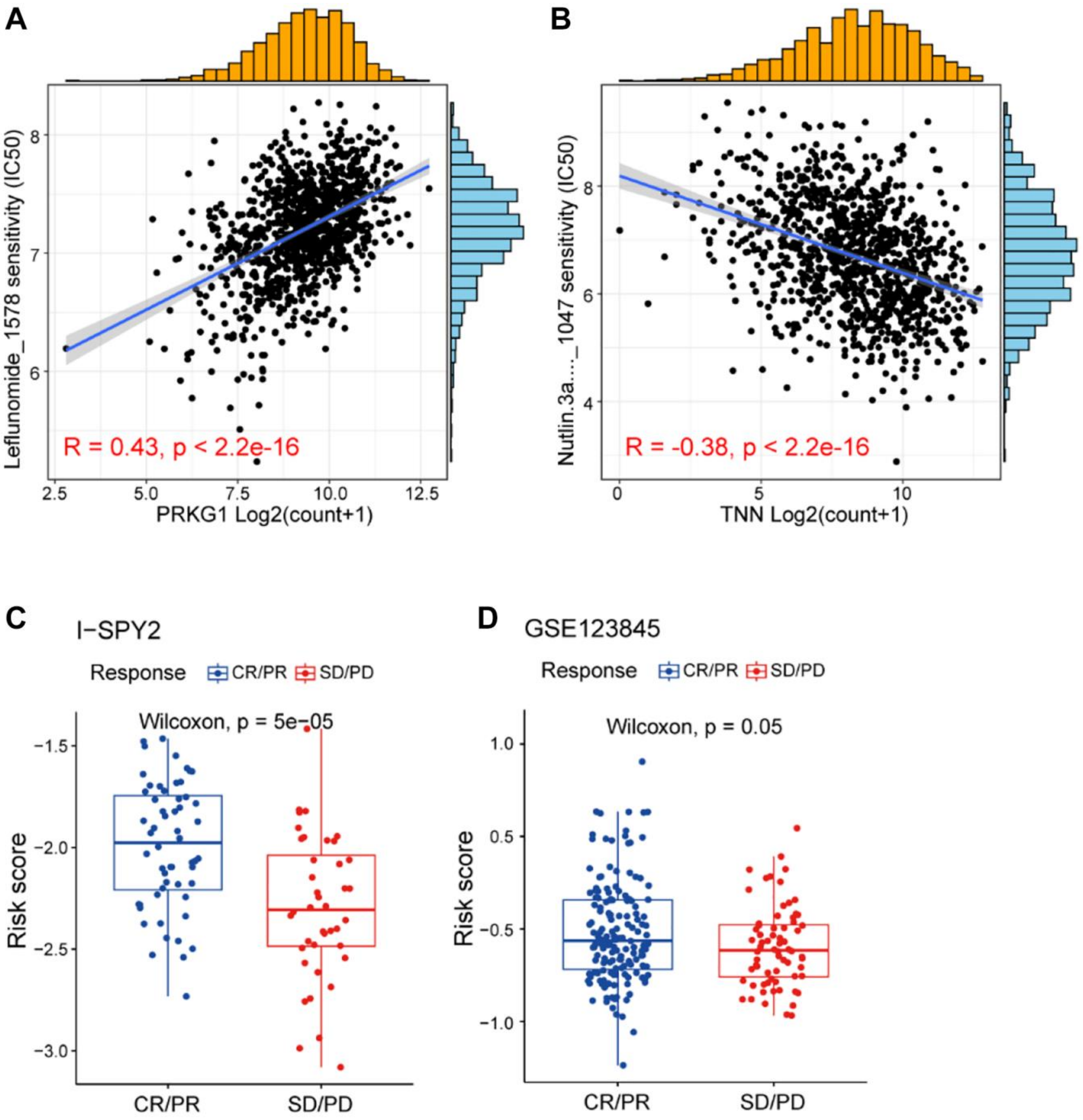

Supplementary Figure 6. High/low-risk group patients differ in drug sensitivity. (**A**) Scatter plots showing the correlation between the expression of PRKG1 and the IC50 of Leflunomide. (**B**) Scatter plots showing the correlation between the expression of TNN and the IC50 of Nutlin.3a .... _1047. (**C**) Boxplot showing the levels of risk score between CR/PR and SD/PD patients in I-SPY2. Paired two-sided Wilcoxon test. (**D**) Boxplot showing the levels of risk score between CR/PR and SD/PD patients in GSE123845. Paired two-sided Wilcoxon test.
