# Supplementary material for: Cancer-associated fibroblast-derived gene signature discriminates distinct prognoses by integrated single-cell and bulk RNA-seq analyses in breast cancer
Source: Aging (Albany NY). 2024 May 9;16(9):8279–305. doi: 10.18632/aging.205817 (PMC11132004; doi:10.18632/aging.205817)
Supplement: Supplementary Table 3 [file aging-16-205817-s003.pdf]

SUPPLEMENTARY TABLES

Supplementary Table 3. A list of significantly prognostic genes identified by univariate Cox regression analysis.

Gene	HR	CI5	CI95	*P*-value
CXCL14	0.92	0.86	0.98	0.008001475
WLS	0.88	0.79	0.99	0.028963359
SDC1	1.21	1.05	1.39	0.009853082
TNN	0.89	0.82	0.96	0.001691972
PDLIM4	0.87	0.77	0.99	0.030797728
ITPRIPL2	1.15	1	1.32	0.047845533
EMP1	1.23	1.05	1.43	0.009450169
CERCAM	1.24	1.03	1.49	0.020515589
MARVELD1	1.24	1.04	1.48	0.017340631
CYTH3	1.23	1.02	1.49	0.027050121
PRKG1	1.16	1.01	1.33	0.030374849
XG	1.15	1.02	1.3	0.026768892
